# Optimization of mucilage extraction from *Ximenia americana* seed using response surface methodology

**DOI:** 10.1016/j.heliyon.2022.e08781

**Published:** 2022-01-19

**Authors:** Asfawosen Mamo Bazezew, Shimelis Admassu Emire, Mulugeta Teamir Sisay, Paulos Getachew Teshome

**Affiliations:** a Chemical Engineering Department, Adama Science and Technology University, Adama, Ethiopia; b School of Chemical and Bioengineering, Department of Food Engineering, Addis Ababa Institute of Technology, P.O.B: 1176, Addis Ababa, Ethiopia; c Ethiopian Institute of Agricultural Research, Ethiopia; d Center for Food Science and Nutrition, Addis Ababa University, Ethiopia

**Keywords:** Cultivar, Response surface method, Yield, Model, Water holding capacity

## Abstract

*Ximenia americana* is a wild edible fruit essential for human consumption due to its high nutritional and phytochemical constituents with significant antioxidant activity. The fruit seed has high potential in its mucilage content. The present study aimed at optimization of mucilage extraction from the Ethiopian cultivar of *Ximeina americana* fruit seed. The response surface methodology based on a central composite rotatable design was used for the optimization of aqueous extraction of mucilage. The extraction temperature (50–80 °C), time (1.5–4 h), and water-to-seed ratio (20:1–40:1 v/w) were identified as the major factors influencing mucilage yield, water holding capacity, and protein content. Water to seed ratio and time showed significant (*p* < 0.01) interaction effect on yield. Interactions of water to seed ratio with time and temperature had significant effects (*p* < 0.05) on the protein content. Water holding capacity of the mucilage was significantly (*p* < 0.05) affected by the interaction between temperature and time. Optimum extraction processing conditions were obtained to be extraction temperature of 65.06 °C, time of 1.5 h and water to seed ratio of 37.62:1 v/w. The response variables at this operating conditions were found to be extraction yield of 17.31 %, water holding capacity of 11.48 g/g and protein content of 1.75 %. The result demonstrated that the *X. americana* seed mucilage could be used as a new source of additives in the dairy industry as a fat replacer due to its potent water holding capacity.

## 1 . Introduction

Mucilage is natural biopolymer commonly obtained from variety of plants and their parts, with the plant seed shared the upper hand (Tosif et al., 2021). Nowadays, awareness of biopolymers are gaining attention because of their broad application in the food industry as film coating, emulsifier, binder, and fat replacer (Ma et al., 2020). The demand for plant based mucilage is rising to be used in different food industries as active ingredients due to safety, availability and low cost (Singh and Barreca, 2020).

*Ximenia americana* is one of the indigenous wild edible fruit to Ethiopia. It belongs to Olacaceae family fruit which is essential for human consumption due to its high nutritional and phytochemical constituents with significant antioxidant activity (Bazezew et al., 2021). The fruit has economic importance in manufacturing oils, cosmetics, medicinal tablets and food ingredients (Getachew and Desta, 2021). When the fruit seed is soaked in water, it forms sticky and viscous mucilaginous solution that could have the potential for mucilage resource. Thus, it was hypothesized that *X. americana* seed mucilage could have considerable importance in improving the food properties and to be used as a new source of food additive for numerous food products. The mucilage is found adhered to the fruit seed and it might yield significant quantity upon the proper extraction method.

Aqueous extraction is one of the most frequently used extraction method for various plant seeds (Hung and Lai, 2019). Cultivar type and extraction conditions are important factors that influence the extraction process, resulting in a wide range of yields and functional properties (Dick et al., 2019). Temperature, water to seed ratio, pH, salt content, solvent nature, and extraction duration are independent variables that affect the mucilage extraction process (Wu et al., 2007).

The preliminary test conducted by the authors on parameter screening experiments demonstrated that extraction temperature, extraction time, and water-to-seed ratio could have notable effect on quality and quantity of mucilage from *X. americana* seed. These factors may have independent or interactive effects with each other. Response surface method (RSM) has been known to be an efficient tool for the optimization of processing conditions when the factors have interactive effect on the chosen responses (Hung and Lai, 2019). This tool used for minimizing the cost and time for large experimental runs (Myers and Montgomery, 1995).

Various scholars studied the aqueous mucilage extraction conditions using RSM for *Hyptis suaveolens* (Morales-Tovar et al., 2020), *Salvia hispanica L*. (Orifici et al., 2018), *Plantago major* seed (Behbahani et al., 2017) and quince seed (Jouki et al., 2014b). They reported that optimization method using RSM for multiple operating conditions was effective on determining the optimal experimental conditions. To the best of our knowledge, no study has been conducted on the optimization of mucilage extraction from *X. americana* seed. Therefore, the objective of this research was to optimize the process conditions for the extraction of mucilage from *X. americana* seed.

## 2 Materials and methods

### 2.1 Plant material collection

The *X. americana* fruit was harvested from Arbaminch area, Ethiopia, located at 5°58′57.04″ N and 37°32′20.4″ E, an altitude of 1269 m above sea level. The fruit was peeled manually and the seed was separated. Then, the seed was ground using mortar and pestle and stored in cool and dry place until the extraction was conducted.

### 2.2 Mucilage extraction

Mucilage extraction was done according to (Jouki et al., 2014a). Briefly, the ground seed samples were mixed with distilled water (99 %) (water to seed ratio of 20:1 to 40:1 (v/w) in the flask. The water was preheated to the desired temperature (50–80 °C) using adjustable water bath (SHA-C, China). The seed slurry was mixed throughout the extraction time (1.5–4 h). The seed suspension was filtered using a fine cloth to remove the remaining small particles and centrifuged with high speed centrifuge (800 centrifuge, China) at 4000 rpm for 8 min. The supernatant was collected and the ethanol 95 % (v/v) was added at the ratio 1:2 to precipitate the mucilage overnight. The precipitate was then collected and dried at 40 °C in a vacuum oven (Model: 4567, Kimya Pars Co., Iran) until it reached a consistent weight. The extraction yield was calculated as the ratio of weight of mucilage obtained after extraction relative to the sample weight used (Alpizar-Reyes et al., 2017).

(1) Yield ​(%)=(Weight ​of ​dried ​mucilage)/(Sample ​weight)×100

### 2.3 Water holding capacity

The determination of Water Holding Capacity (WHC) was conducted according to (Ghribi et al., 2015). Mucilage dispersions 1% (w/v) was prepared and placed in previously weighted centrifuge tubes. The mixture was vortexed for 2 min. Then, the dispersions was centrifuged with high speed centrifuge (model: 800 centrifuge, China) at 2200 rpm for 15 min. The supernatant was removed, and the remaining residue was weighed again.

(2) WHC ​(g/g)=(Water ​absorbed ​weight)/(Sample ​weight)

### 2.4 Protein content

The total nitrogen content of sample was determined using Kjeldahl method AOAC, 2000 No. 920.87. Briefly, 0.5 ± 0.05 g of dried mucilage was digested in an auto-digester by heating in the presence of 20 mL of concentrated sulphuric acid containing two copper catalyst tablets at 420 °C for 2 h. The digest was filtered into a 250 mL volumetric flask and the solution made up to mark with distilled water and connected with distillation (Automatic distillation unit, VELP Scientifica srl, Usmate Velate (MB), Italy). Ammonia was steam distilled from the digest to which had been added 50 mL of 45 % sodium hydroxide solution. 150 mL of the distillate was collected in a conical flask containing 100 mL 0.1N HCl and methyl red indicator. The ammonia that was distilled into the receiving conical flask reacted with the acid and the excess acid in the flask was estimated by back titration against 2.0 M NaOH with color change (end point). Determinations were made on all reagents alone (blank determinations). Protein content was calculated by multiplying the total nitrogen with a factor of 6.25 (Muthai et al., 2017).

### 2.5 Experimental design and statistical analysis

Though many factors affect the extraction process of *X. americana* seed mucilage, the parameter screening experiments demonstrated that extraction temperature, time, water to seed ratio and pH were were found to be the major factors. To induce natural extraction of mucilage, pH was not considered for the optimization of extraction process. The remaining factors had negligible effect on the extraction process of mucilage from *X. americana* seed.

To establish the ranges that influence the extraction process, preliminary tests were conducted using single factor analysis. The other two independent variables were held constant while one independent variable was studied. The response criteria were maximum mucilage yield and water holding capacity with low protein content. When the effect of extraction temperature (15–110 °C) investigated, the extraction time and water to seed ratio kept constant at 2.75 h and 50:1, respectively. To study the effect of extraction time (0.5 – 6h), the water to seed ratio and extraction temperature were kept constant at 50:1 and 60 °C. To investigate the effect of water to seed ratio (10:1 to 100:1), the extraction temperature and time fixed to 60 °C and 2.75 h, respectively.

The extraction factor values corresponding to each independent variable were chosen based on the results of the single factor preliminary test, with extraction time ranging from 1.5 to 4 h, temperature ranging from 50 to 80 °C, and water to seed ratio ranging from 20:1 to 40:1 (v/w).

The extraction temperature, T, water to seed ratio, W and time, t were assessed using Design-Expert version 7.0 (Minneapolis, USA). The data for the experiment was designed using a central composite rotatable design (CCRD) (Table S1). The minimum level of each factor was set in place of - alpha (−1.68) and the maximum level of each factor was set in place of + alpha (+1.68) values in CCRD.

Analysis of variance (ANOVA) was used as an evaluation tool for the significant terms for each responses in different models. The F- statistics resulting from the data should be less than 5%. The coefficient of determination (R^2^), adjusted-R^2^ and predicted-R^2^ were used as criteria to check the model adequacy. Adequate model need to have a large R^2^, adj-R^2^ and pre-R^2^ (Kumar et al., 2017).

The independent variables for the extraction temperature, water to seed ratio, and extraction time were linked to the coded variables (T_i_, W_i_ and t_i_, i = 1, 2 and 3; β_0_ is a constant; β_1_, β_2_, β_3_ are linear coefficients; β_12_, β_13_, β_23_ are interaction coefficients, and β_11_, β_22_, β_33_ are quadratic coefficients) using a second order polynomial.

(3) $$Y=\beta_{0}+\beta_{1}\text{T}+\beta_{2}\text{ ​W}+\beta_{3}\text{t}+\beta_{11}{\text{T}}^{2}+\beta_{22}{\text{W}}^{2}+\beta_{33}{\text{t}}^{2}+\beta_{12}\text{TW}+\beta_{13}\text{Tt}+\beta_{23}\text{Wt}$$

A numerical optimization method was used for optimization of multiple dependent variables. The dependent variables were kept either minimized or maximized whereas the independent variables were fixed in ranges.

### 2.6 Validation of developed models

The model's adequacy was validated using the determined optimum aqueous extraction process conditions.

## 3 Result and discussion

### 3.1 Model fitting for response surface

The experimental result from the complete design of the central composite rotatable design (CCRD) for the factors (temperature, water to seed ratio and time) and responses (yield, protein content and water holding capacity) is shown in Table 1.

**Table 1:** Experimental design with the observed responses of yield, protein and water holding capacity of mucilage.

Run	T	W	t	Response Y		
	Temperature, °C	Water: seed, v/w	Time, h	Yield (%)	Protein content (%)	Water holding capacity (%)
1	50	30	2.75	10	1.2	4.45
2	65	30	2.75	19.62	2.84	9.81
3	74	24	2	11	2.7	11.38
4	65	30	2.75	18.8	2.79	10.51
5	65	30	4	20.15	2.85	11.21
6	74	36	4	16.5	2.9	12.39
7	65	20	2.75	12	2.7	9.71
8	65	30	2.75	18.41	2.91	10.81
9	65	40	2.75	17	3.15	11.74
10	65	30	1.5	15.32	1.97	10.48
11	56	36	2	17.05	1.64	8.27
12	80	30	2.75	10.35	2.2	8.84
13	74	24	4	15.40	2.85	9.85
14	65	30	2.75	19.25	2.76	10.19
15	65	30	2.75	18.9	2.95	10.59
16	56	34	3.49	14.43	2.74	9.64
17	56	24	2	11.21	1.79	7
18	56	24	3.49	12.5	1.92	8.43
19	74	36	2	14.59	2.31	11.95
20	65	30	2.75	19.97	3.24	10.58

T: Temperature °C, W: water: seed ratio v/w, t: time h.

Multiple regression analysis and ANOVA were used for fitting the model and to evaluate the statistical significance of the terms (Table S2). The experimental data evaluation indicated that second-order polynomial model (Eq. 3) can adequately explain the data (p < 0.0001) sufficiently. Therefore, it could be the most fitting model for the three dependent variables.

The ANOVA analysis in this study showed that lack of fit was not significant (p > 0.05) for the chosen variables, which indicated that this model was adequately precise for predicting the responses.

From Table 2, it can be observed that the R^2^ values for the response variables were 0.98, 0.96 and 0.96 for extraction yield, protein content and water holding capacity, respectively. This result explains the model suitability for the response variables.

**Table 2:** Regression coefficients for the fitted quadratic polynomial model and analysis of variance for the experimental results of yield (%), protein (%) and water holding capacity (%).

Sources	Yield (%)	Protein (%)	Water holding capacity (%)
Intercept	19.15	2.91	10.4
Linear			
T- Temperature	0.21^ns^	0.32^a^	1.44^a^
W-Water to seed ratio	1.53^a^	0.08^ns^	0.66^b^
t- Time	0.96^b^	0.25^b^	0.22^ns^
Interaction			
TW	0.38^ns^	−0.13^d^	0.08^ns^
Tt	0.96^c^	−0.06^ns^	−0.49^d^
tW	−0.80^c^	0.18^c^	0.24^ns^
Quadratic			
T^2^	−3.12^a^	−0.42^a^	−1.22^a^
W^2^	−1.59^a^	0.01^ns^	0.22^ns^
t^2^	−0.45^d^	−0.17^c^	0.26^ns^
R^2^	0.98	0.96	0.96
Adj-R^2^	0.96	0.92	0.93
Pred-R^2^	0.89	0.85	0.78
*P*- value	<0.0001	<0.0001	<0.0001
*F*- value	56.20	27.31	31.22
C. V. %	4.25	5.98	4.76
Lack of fitness	1.28	1.5	2.46

^a^ Significant at P < 0.0001; ^b^ Significant at P < 0.001;^c^ Significant at P < 0.01; ^d^ Significant at P < 0.05 and ^ns^ Non-significant.

It's important to note that as the number of variables in the model increases, the R^2^ value increases as well, regardless of the statistical significance of the additional variables. As a result, higher R^2^ values may not always explain model suitability. As a result, assessing the model's adequacy requires checking an adj-R^2^, which must be greater than 90 %. The adj-R^2^ values in this study for extraction yield, protein content and water holding capacity were 0.96, 0.92 and 0.93, respectively. This result indicated that the higher adj-R^2^ described the model with non-significant terms.

The coefficient of variation (CV) expresses the tendency for the data to be non-continuous. As a rule of thumb, the CV should not be greater than 10 %. A higher CV denotes a greater average difference in average results, and thus does not produce adequate response models (Kumar et al., 2017). From this study, the coefficient of variation for extraction yield, protein content and water holding capacity were 4.25, 5.98 and 4.76, respectively. The result for the coefficient of variation described better precision and suitability of the experiments (Table 2).

According to the multiple regression and ANOVA analysis, the models utilized in this study were capable of defining operating conditions for mucilage extraction from .*X. americana* seed.

### 3.2 Effect of independent variables on yield

The correlation between independent and dependent variables was represented in the three-dimensional response surface plot generated by the model. Within the experiment under consideration, data was obtained by fixing the two variables at constant values or center points and altering the remainder variable.

The presence of interaction between the factors was revealed by the response surface investigation. The ANOVA model of the extraction yield showed the significance (p < 0.05) of linear effect of water to seed ratio and extraction time except temperature (Table 2). The coefficient of all three quadratic effects of the three independent variables were significant (*p* < 0.05) for the extraction yield. This could be due to the self-interaction of parameter on the yield of mucilage.

Among the interaction effects, extraction temperature vs. time and water to seed ratio vs. time had significant effects on extraction yield. The result also illustrated water to seed ratio and time (*p* < 0.01) showed significant interaction. This could be owing to the combined effect of one parameter on the other terms for the yield of mucilage.

The model indicated that yield was mainly influenced by water: seed ratio and time, and to a slighter degree by temperature.

**Figure 1:**
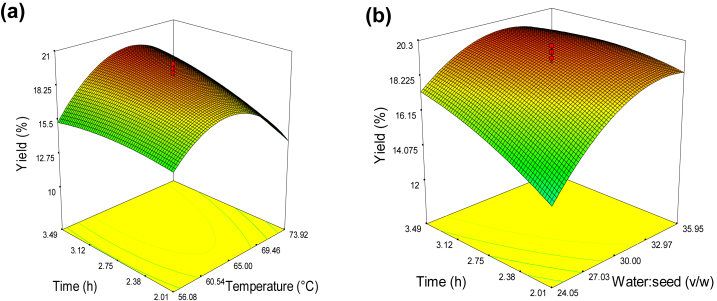
Response surface for the effect of independent variables on extraction yield of *X. americana* seed mucilage: (a) time and temperature (water: seed 30:1), and (b) extraction time and water: seed ratio (temperature: 65 °C).

The correlation between the temperature and time at fixed water to seed ratio is shown in Figure 1a. The extraction yield increased up to a temperature of 69 °C and begins to decline. This might be due to temperature effect that accelerate the extraction ability of the solvent, water in this case. Temperature allows water to penetrate the seeds more easily, allowing the mucilage to be simply dissolved and released by decreasing the mucilage viscosity in the seed. As a result, the amount of mucilage extracted had increased. However, the decline in the extraction yield after a temperature of 69 °C could be related to mucilage hydrolysis at higher extraction temperatures. The yield of mucilage was also found to increase with increasing extraction time. This might be due to increased reaction time between the seed and water. Similar results were observed for mucilage extraction from Basil seeds (Nazir et al., 2017).

The effect of water to seed ratio and extraction time on yield is shown in Figure 1b. The increase in water to seed ratio promoted the mucilage yield increment. The significance of water in ensuring mass transfer during extraction was presumably the reason for the considerable dependency of mucilage yield on the water to seed ratio. The accumulation of water in the endosperm led to the binding of water soluble components. As a result, it increased the extraction yield. The higher water content makes the slurry less sticky, providing a more efficient extraction of mucilage (Amid and Mirhosseini, 2012). The water to seed ratio contribute for the efficiency of extraction yield of mucilage. Therefore, it is necessary to optimize the water usage during mucilage extraction (Souza et al., 2020). Increasing extraction time had also significant effect on the mucilage yield. This might be due to time exposure allowing better mass transfer and penetration of solvent into seed, which resulted higher yield.

The dependence of the extraction yield on the independent factors can be rated as: water: seed ratio > extraction time > extraction temperature. This result disagree with the report of Hung and Lai (2019) and Campos et al. (2016), who studied mucilage extraction from *Basella alba* and *Lepidium perfoliatum* seed, respectively. This could be due to variation in plant seed and extraction conditions.

The regression equation using the coded levels of the independent variables for mucilage yield was as follows:

(4) $$Y=19.15+0.21\text{T}+1.53\text{W}+0.96\text{t}-0.38\text{TW}\;+0.96\text{Tt}-0.80\text{Wt}-3.12{\text{T}}^{2}-1.59{\text{W}}^{2}-0.45{\text{t}}^{2}$$

Where; Y- mucilage yield (%), T- temperature (°C), W- water to seed ratio (v/w), t- time (h).

### 3.3 Effect of independent variables on protein content

The presence of lower protein content in the mucilage is associated with its purity. This might depend on the inherent presence of structural proteins and enzymes in the mucilage. The extraction time and temperature had significant (*p* < 0.0001) linear effect, whereas no significant effect was observed on varying the water to seed ratio (Table 2). The extraction time and temperature had significant quadratic effect *(p* < 0.05) and (*p* < 0.0001), respectively. From the interaction effect, water to seed ratio and time as well as temperature and water to seed ratio had a significant effect (*p* < 0.05) on the protein content. Protein content's reliance on independent factors could be rated as: extraction temperature, time and water: seed ratio.

**Figure 2:**
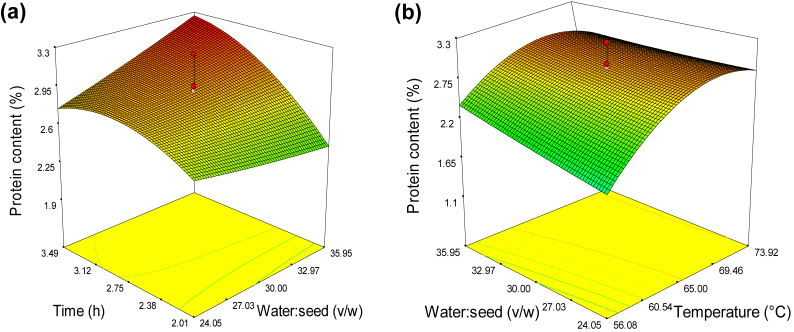
Response surface for the effect of independent variables on protein content of *X. americana* seed mucilage: (a) time and water to seed ratio (temperature: 65 °C), and (b) water: seed ratio and extraction temperature (time: 2.75 h).

The effect of water to seed ratio and time on protein content is illustrated in Figure 2a. The protein content rose as the extraction time was increased up to a certain point. Increasing the extraction time allows more solvents to seep into the seeds. This aids in the dissolution of proteins that are to be dispersed out of the seeds. In this study, the protein content of *X. americana* seed ranged from 1.20 % to 3.24 %, depending on the extraction conditions. Little effect has been observed on the water to seed ratio on protein content. The result from this study was within the range as compared to the commercial xanthan gum (2.12%) (Razavi, 2019).

The protein content increased with temperature as depicted in Figure 2b. The increment of protein content with temperature up to certain value could be explained by the higher mass transfer rate. Increasing the extraction temperature, on the other hand, resulted in a decrease in protein content, most likely due to thermal denaturation of protein at higher temperatures. Similar trend was reported for quince seed mucilage (Jouki et al., 2014b). The water to seed ratio also showed a slight increase in the protein content. The increased protein content in the mucilage with water to seed ratio increment might be associated with intrinsic protein content of mucilage and the crushed hard seed core incorporated during extraction.

The regression equation using the coded levels of the independent variables and response variable was shown as follows:

(5) $$Y\;=2.91+0.32T+0.08\;W+0.25\;t-0.13\;TW-0.061\;Tt+0.18\;Wt-0.42T^{2}+0.013W^{2}-0.17\;t^{2}$$

Where; Y- Protein content (%), T- temperature (°C), W- water to seed ratio (v/w) and t- time (h).

The independent factors which yield lowest protein content were temperature (50 °C), extraction time (2.75 h) and water to seed ratio of (30:1), respectively. Andrade et al. (2020) reported similar observation for taro mucilage.

### 3.4 Effect of independent variables on water holding capacity (WHC)

Water Holding Capacity refers to a food's ability to hold water under various forces (WHC). It is also known to modify the texture and viscosity of food products (Singh et al., 2001). Table 2 lists the significance (P < 0.001) of the linear influence of water to seed ratio and temperature. The effect of the independent variable on WHC demonstrated that the temperature significantly (*p* < 0.001) impacted the mucilage water holding capacity with the quadratic model. The interaction between temperature and time was also significant (*p* < 0.05).

**Figure 3:**
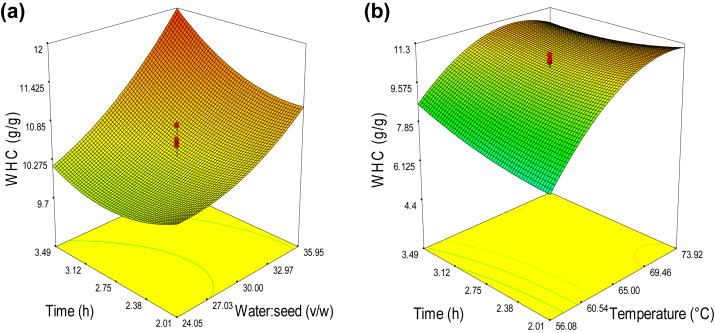
Response surface for the effect of independent variables on the water holding capacity of *X. americana* seed mucilage: (a) time and temperature (water: seed 30:1), and (b) extraction time and water: seed ratio (temperature: 65 °C).

The three-dimensional surface plots (Figure 3a) indicated that the water holding capacity increased as the temperature increased up to 69.46 °C and then decreased in parabolic manner. A minor increment was also observed in water holding capacity with time. The difference in water holding capacity under different extraction conditions could depend on the polar hydroxyl groups and the degree of hydrodynamic interactions (Koocheki et al., 2012).

The surface plot for the time and water to seed ratio effect on water holding capacity was shown in Figure 3b. There was a substantial increase in water holding capacity as the water to seed ratio increases. Similar findings were reported by other scholars (Behbahani et al., 2017b). The trajectory of the time effect, on the other hand, indicated a decline in water holding capacity until a specific value was reached, after which it proceeded to rise and reached maximum point of 3.49 h. This might be due to the contribution of fine structure with plenty of small pores in mucilage. Similar observation was reported for *Arabinoxylan* and *rhamnogalacturonan* mucilage (Kamel et al., 2020).

The regression equation using the coded levels of the independent variables and response variable was as follows:

(6) $$Y=10.4+1.44T+0.66\;W+0.22\;t+0.079\;TW-0.49\;Tt+0.24\;Wt-1.22T^{2}+0.22W^{2}+0.26t^{2}$$

Where; Y- Water holding capacity (g/g), T- temperature (°C), t- water to seed ratio (v/w) and W- time (h).

The higher water holding capacity (12.39 g/g) was obtained at independent variables of temperature 73.92 °C, time 3.49 h, and water to seed ratio of 35.95:1. The higher water holding capacity of this mucilage makes it a good candidate to be used as fat replacer in improving the physicochemical properties of low fat or fat free food products.

### 3.5 Model adequacy checking

To ensure that the fitted model offers a sufficient estimate of the real system, it must be tested for model adequacy. It is not possible to proceed with the optimization of the fitted response surface without first verifying the model for acceptable fit, as this would likely result in poor and unclear results (Samavati, 2013). The residual from least squares fit (Figure S1) is an important criteria in confirming model adequacy (Borror et al., 2002). Model adequacy can be checked with the use of residual from least squares fit (Rashid et al., 2019).

Using a normal probability plot of the residuals, the normality of the three responses was confirmed (Figure S2). The straight line on the normal plot of residuals confirmed normality assumption sufficiently. The assumption of the normality was also satisfactorily satisfied using the residual plot vs. the predicted response. The general rule is that the random residual scatter on the display means that the variance of original observation is constant for all Y values. As a result, the empirical model was found to be acceptable for describing the yield, water holding capacity, and protein content of *X. americana* seed mucilage by response surface.

### 3.6 Optimization and model verification

The goal of the optimization analysis for *X. americana* seed mucilage extraction was to get the highest extraction yield and water holding capacity with the minimum amount of protein. The optimal condition produced a maximum mucilage yield of 17.31 %, a water holding capacity of 11.48 g/g, and a minimum protein content of 1.75 % (Table 3). Souza et al. (2020) reported higher mucilage yield for *psyllium* mucilage than the current study. However, it was found that (Hung and Lai, 2019) observed lower mucilage yield for *basella alba* mucilage compared to the present study. Orifici et al. (2018) also concluded that the mucilage yield was 11.6 % under optimum processing conditions of temperature at 85 °C, seed to water ratio 1:31 and extraction time of 2 h for chai seed.

**Table 3:** Predicted optimum conditions for extraction of *X. americana* seed mucilage.

Variables	Minimum	Maximum	Optimum
Temperature	50	80	65.06
Time	1.5	4	1.5
Water: seed	20:1	40:1	37.63:1

The extraction experiments were conducted by comparing the model prediction with the optimum levels achieved by the RSM optimization, to test the adequacy of the response surface model for the optimum anticipated values (extraction time 1.5 h, water to seed ratio 37.62:1 and extraction temperature 65.06 °C). Table 4 shows validation results of the anticipated and mean of experimental values for extraction yield, protein content, and water holding capacity. These values confirmed that both the experiments and predicted values agree with each other. The non-significant variation of both values confirms the validity and adequacy of the predicted model (Eq. 3). The results indicated that the model used can identify operating conditions for extraction of mucilage from *X. americana* seed.

**Table 4:** Predicted and experimental values of the responses obtained at optimum conditions.

Response	Predicted value	Experimental value^a^
Extraction yield (%)	17.31	16.58 ± 0.84
Protein content (%)	1.75	1.92 ± 0.17
Water holding capacity (g/g)	11.48	12.06 ± 0.69

a Mean (n = 3).

## 4 Conclusions

The effects of processing conditions for the extraction of mucilage from *Ximenia americana* seeds were studied using RSM based on CCRD. The ANOVA analysis showed the suitability of RSM for response variables. Temperatures as high as 69 °C had a positive correlation with yield and water holding capacity in this study. Water to seed ratio showed the leading effect on the yield of mucilage. The water holding capacity was positively affected by water to seed ratio and temperature. However, the opposite correlation was observed for the effect of time on water holding capacity. The increase in temperature and time had a significant increasing effect on protein content, but the water to seed ratio showed slight change. The optimized response surface extraction conditions for temperature, time and water to seed ratio were 65.06 °C, 1.5 h, and 37.62:1, respectively. In this study, the Ethiopian cultivar of *X. americana* seed showed high potential to be used as a source of mucilage. With further evaluations of the bioactive compounds, the chemical constituents responsible for the medicinal effects and bioavailability of the active substances, *X. americana* seed mucilage could be used as a food ingredient in food industries.

## Declarations

### Author contribution statement

Asfawosen Mamo Bazezew: Conceived and designed the experiments; Performed the experiments; Analyzed and interpreted the data; Wrote the paper.

Shimelis Admassu Emire, Mulugeta Teamir Sisay & Paulos Getachew Teshome: Conceived and designed the experiments; Contributed reagents, materials, analysis tools or data; Wrote the paper.

### Funding statement

This work was supported by the Addis Ababa University and Adama Science and Technology University.

### Data availability statement

Data included in article/supp. material/referenced in article.

### Declaration of interests statement

The authors declare no conflict of interest.

### Additional information

No additional information is available for this paper.

## Appendix A Supplementary data

Supplementary files.docx Supplementary files.docx
