# Supplementary material for: Optimization of mucilage extraction from Ximenia americana seed using response surface methodology
Source: Heliyon. 2022 Jan 19;8(1):e08781. doi: 10.1016/j.heliyon.2022.e08781 (PMC8800025; doi:10.1016/j.heliyon.2022.e08781)
Supplement: Supplementary files.docx [file mmc1.docx]

**Supplementary files**

**Table S1.** Experimental design with actual and coded levels for the Temperature ℃, water: seed ratio v/w and time h.

|  |  |  | Run |  |  |  |
| --- | --- | --- | --- | --- | --- | --- |
|  | Actual level |  |  |  | Coded level |  |
| Run | Temperature,℃ | Water: seed, v/w | Time, h | T | W | t |
| 1 | 50 | 30 | 2.75 | -1 | 0 | 0 |
| 2 | 65 | 30 | 2.75 | 0 | 0 | 0 |
| 3 | 74 | 24 | 2 | 1 | -1 | -1 |
| 4 | 65 | 30 | 2.75 | 0 | 0 | 0 |
| 5 | 65 | 30 | 4 | 0 | 0 | 1 |
| 6 | 74 | 36 | 4 | 1 | 1 | 1 |
| 7 | 65 | 20 | 2.75 | 0 | -1 | 0 |
| 8 | 65 | 30 | 2.75 | 0 | 0 | 0 |
| 9 | 65 | 40 | 2.75 | 0 | 1 | 0 |
| 10 | 65 | 30 | 1.5 | 0 | 0 | -1 |
| 11 | 56 | 36 | 2 | -1 | 1 | -1 |
| 12 | 80 | 30 | 2.75 | 1 | 0 | 0 |
| 13 | 74 | 24 | 4 | 1 | -1 | 1 |
| 14 | 65 | 30 | 2.75 | 0 | 0 | 0 |
| 15 | 65 | 30 | 2.75 | 0 | 0 | 0 |
| 16 | 56 | 34 | 3.49 | -1 | 1 | 1 |
| 17 | 56 | 24 | 2 | -1 | -1 | -1 |
| 18 | 56 | 24 | 3.49 | -1 | -1 | 1 |
| 19 | 74 | 36 | 2 | 1 | 1 | -1 |
| 20 | 65 | 30 | 2.75 | 0 | 0 | 0 |

T: Temperature ℃, W: water: seed ratio v/w, t: time h.

**Table S2.** The significance of Extraction yield (%), Protein content (%) and Water holding capacity (g/g).

| Source | Variable | Extraction yield | | | Protein content | | | Water holding capacity | | |  |
| --- | --- | --- | --- | --- | --- | --- | --- | --- | --- | --- | --- |
|  |  | DF | SS | *P*-value | DF | SS | *P*-value | DF | SS | *P*-value | |
| Source |  | 9 | 223.19 | <0.0001 | 9 | 5.59 | <0.0001 | 9 | 62.21 | <0.0001 |  |
| Linear effect | T | 1 | 0.61 | 0.26 | 1 | 1.39 | <0.0001 | 1 | 28.17 | <0.0001 |  |
|  | W | 1 | 31.89 | <0.0001 | 1 | 0.086 | 0.079 | 1 | 5.94 | 0.0004 |  |
|  | t | 1 | 12.57 | 0.0003 | 1 | 0.87 | 0.0001 | 1 | 0.63 | 0.12 |  |
| Quadratic effect | T^2^ | 1 | 140 | <0.0001 | 1 | 2.54 | <0.0001 | 1 | 21.56 | <0.0001 |  |
|  | W^2^ | 1 | 36.6 | <0.0001 | 1 | 0.0002 | 0.74 | 1 | 0.69 | 0.10 |  |
|  | t^2^ | 1 | 2.92 | 0.027 | 1 | 0.41 | 0.0017 | 1 | 0.99 | 0.06 |  |
| Interaction effect | TW | 1 | 1.19 | 0.13 | 1 | 0.14 | 0.03 | 1 | 0.05 | 0.64 |  |
|  | Tt | 1 | 7.3 | 0.002 | 1 | 0.03 | 0.27 | 1 | 1.89 | 0.015 |  |
|  | Wt | 1 | 5.12 | 0.006 | 1 | 0.25 | 0.007 | 1 | 0.46 | 0.18 |  |

T: temperature, W: water to seed, t: time.

1. (b)

(c)

**Figure S1.** Internally studentized residuals vs. predicted response for (**a**) mucilage yield, (**b**) water holding capacity and (**c**) protein content.

1. (b)

(c)

**Figure S2.** Normal probability of internally studentized residuals for (**a**) extraction yield, (**b**) protein .content and (**c**) water holding capacity.
